# Supplementary material for: Effects of Dietary Fiber on Growth Performance, Nutrient Digestibility and Intestinal Health in Different Pig Breeds
Source: Animals (Basel). 2022 Nov 25;12(23):3298. doi: 10.3390/ani12233298 (PMC9740264; doi:10.3390/ani12233298)
Supplement: Supplementary file 1 [file animals-12-03298-s001.zip › animals-1962133-supplementary.pdf]

**Table S1.** Primers sequences used for quantitative RT-PCR.

| Gene | Primer sequence (5' –3') | Annealing temperature (°C) | Product size (bp) |
|---|---|---|---|
| β-Actin | F: TGGAACGGTGAAGGTGACAGC<br>R: GCTTTTGGGAAGGCAGGGACT | 60 | 177 |
| *SGLT-1* | F: CCACTTTCCCTATAAAACCTCAC<br>R: CTCCATCAAACTTCCATCCTCAG | 60 | 151 |
| *GLUT-2* | F: CCTGCTTGGTCTATCTGCTGTG<br>R: TTGATGCTTCTTCCCTTTCTTT<br>R: GAGCCCACAAAGAAAAGC | 60 | 156 |
| *FATP-1* | F: GGAGTAGAGGGCAAAGCAGG<br>R: AGGTCTGGCGTGGGTCAAAG | 60 | 208 |
| *ZO-1* | F: CAGCCCCCGTACATGGAGA<br>R: GCGCAGACGGTGTTCATAGTT | 60 | 114 |
| Occludin | F: CTACTCGTCCAACGGGAAAG<br>R: ACGCCTCCAAGTTACCACTG | 60 | 158 |
| Claudin-1 | F: GCCACAGCAAGGTATGGTAAC<br>R: AGTAGGGCACCTCCCAGAAG | 60 | 140 |
| Total bacteria | F: ACTCCTACGGGAGGCAGCAG<br>R: ATTACCGCGGCTGCTGG | 60 | 200 |
| *Lactobacillus* | F: GAGGCAGCAGTAGGGAATCTTC<br>R: CAACAGTTACTCTGACACCCGTTCTTC<br>P: AAGAAGGGTTTCGGCTCGTAAAACTCTGTT | 60 | 126 |
| *Escherichia coli* | F: CATGCCGCGTGTATGAAGAA<br>R: CGGGTAACGTCAATGAGCAAA<br>P: AGGTATTAACTTTACTCCCTTCCTC | 60 | 96 |
| *Bifidobacterium* | F: CGCGTCCGGTGTGAAAG<br>R: CTTCCCGATATCTACACATTCCA<br>P: ATTCCACCGTTACACCGGGAA | 60 | 121 |
| *Bacillus* | F: GCAACGAGCGCAACCCTTGA<br>R: TCATCCCCACCTTCCTCCGGT<br>P: CGGTTTGTCACCGGCAGTCACCT | 60 | 92 |

ZO-1, zonula occludens-1; FATP1, Fatty acid transport protein-1; SGLT1,sodium glucose transport protein-1.
